# Supplementary material for: Mechanistic Investigation of WWOX Function in NF-kB-Induced Skin Inflammation in Psoriasis
Source: Int J Mol Sci. 2023 Dec 21;25(1):167. doi: 10.3390/ijms25010167 (PMC10779412; doi:10.3390/ijms25010167)
Supplement: Supplementary file 1 [file ijms-25-00167-s001.zip › ijms-2770340-supplementary.pdf]

Supplementary Information

Mechanistic Investigation of WWOX Function in NF-kB-induced Skin Inflammation in Psoriasis

Min Jeong Shin [1,†], Hyun Sun Kim [2,†], Pyeongan Lee [2], Na Gyeong Yang [1], Jae Yun Kim [1], Yun Su Eun [1], Whiin Lee [1], Doyeon Kim [3], Young Lee [3], Kyung Eun Jung [3], Dongkyun Hong [3], Jung Min Shin [3], Sul Hee Lee [5], Sung Yul Lee [1], Chang Deok Kim [3,4,*] and Jung Eun Kim [1,*]

[1] Department of Dermatology, College of Medicine, Soonchunhyang University Cheonan Hospital, Cheonan, 7 31151, Republic of Korea

[2] Department of Dermatology, Soonchunhyang University Graduate School of Medicine, Asan, 31538, Republic 9 of Korea

3 Department of Dermatology, School of Medicine, Chungnam National University Hospital, Daejeon, 35015, 11 Republic of Korea

4 Department of Medical Science, School of Medicine, Chungnam National University, Daejeon, 35015, Repub-13 lic of Korea

5 Department of Dermatology, College of Medicine, Soonchunhyang University Bucheon Hospital, Bucheon, 15 14584, Republic of Korea

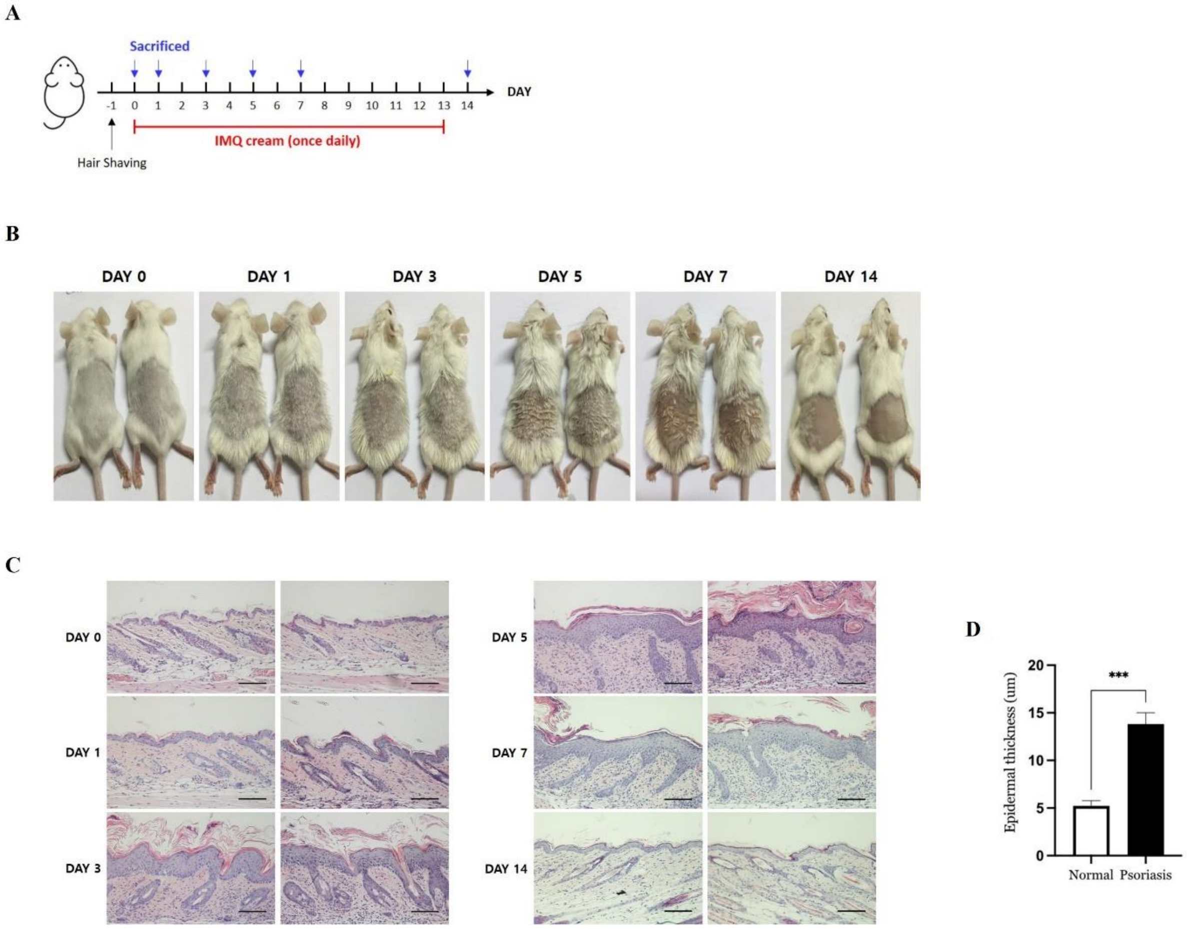

Figure S1. (A) Timeline of the imiquimod-induced psoriasis mouse model. (B) Representative images of the back skin from the IMQ-induced mice model at different time points. (C) Representative Hematoxylin and Eosin staining findings of the back skin from the IMQ-induced mice model at different time points. (n = 2, Magnification: 200X, Scale bar = 100μm) (D) Histogram of Epidermal thickness of day 5. Data are presented as mean ± SEM (n = 5). Student's t-test was performed for statistical analysis. * $P < 0.05$.

WWOX - Chronic plaque psoriasis: lesional and non-lesional skin punch biopsies
Annotation: WWOX, WW domain-containing oxidoreductase
Organism: Homo sapiens
Reporter: GPL570, 221147_x_at (ID_REF), GDS5392, 51741 (Gene ID)
DataSet type: Expression profiling by array, transformed count, 8 samples
ID: 124567731

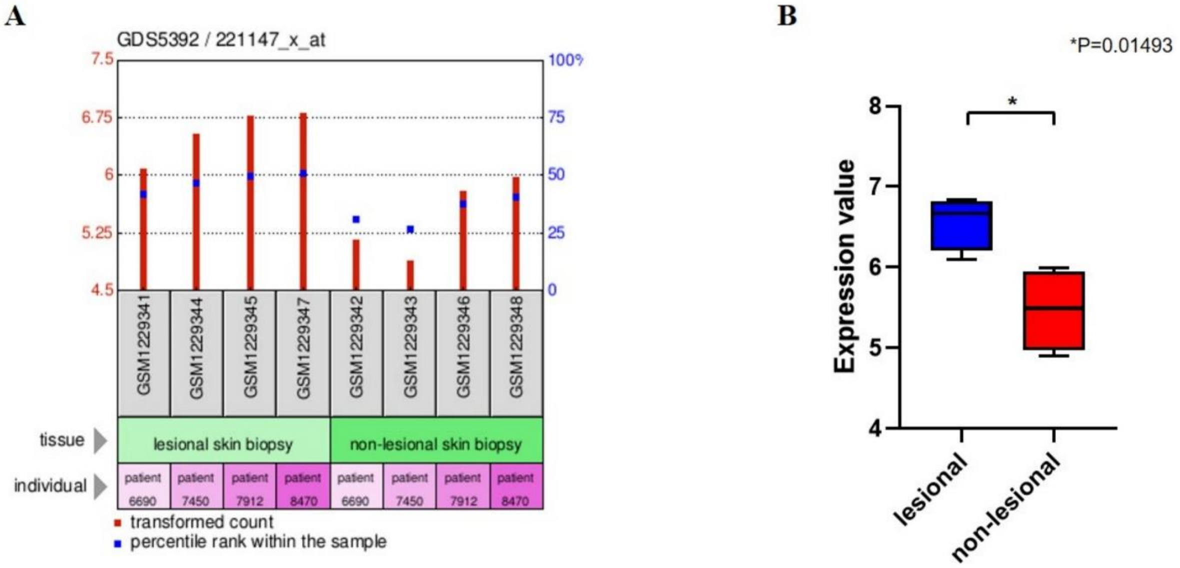

Sample	Title	Value	Rank
GSM1229341	Gudjohnsson_001_6690_PP	6.10035	42
GSM1229344	Gudjohnsson_004_7450_PP	6.54777	47
GSM1229345	Gudjohnsson_005_7912_PP	6.78228	50
GSM1229347	Gudjohnsson_007_8470_PP	6.83121	51
GSM1229342	Gudjohnsson_002_6690_PN	5.17769	31
GSM1229343	Gudjohnsson_003_7450_PN	4.90249	27
GSM1229346	Gudjohnsson_006_7912_PN	5.80526	38
GSM1229348	Gudjohnsson_008_8470_PN	5.98751	41

Figure S2. (A) Processed microarray data were obtained from Gene Expression Omnibus. (B) Transcript levels in lesional area were compared to levels in non-lesional area.

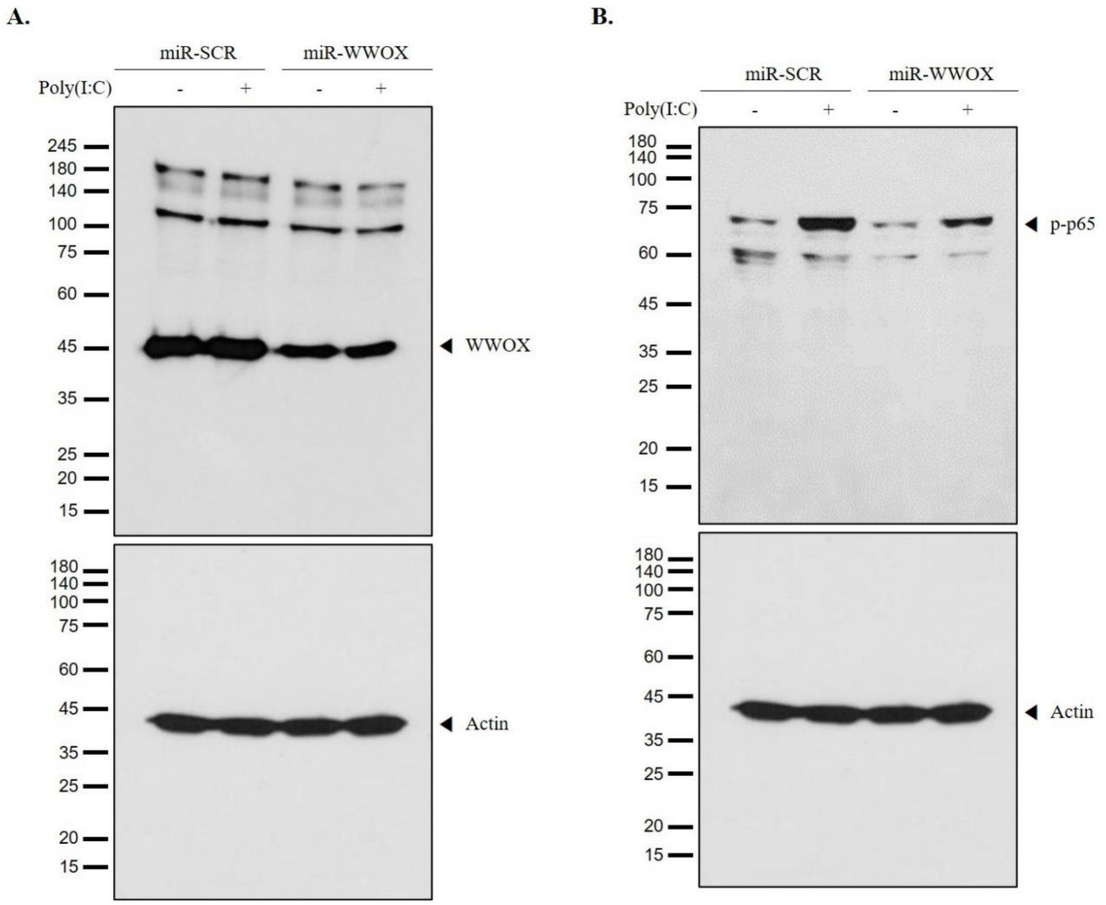

Figure S3. Uncropped data for Figure 2.

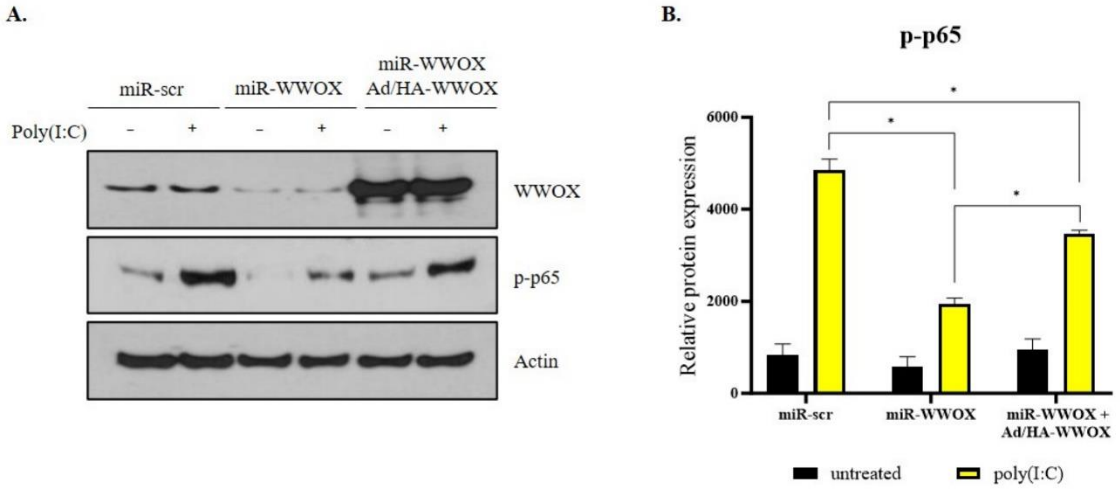

Figure S4. Rescue Experiment by overexpression of WWOX in SV-keratinocyte. (A) The rescue experiment of Western blot showed that overexpression of WWOX increased p-p65 (NF-kB marker), which was decreased by WWOX knockdown. (B) Histogram of relative protein expression. Data are presented as mean ± SEM (n = 3). Student's t-test was performed for statistical analysis. $^{*} P < 0.05$.

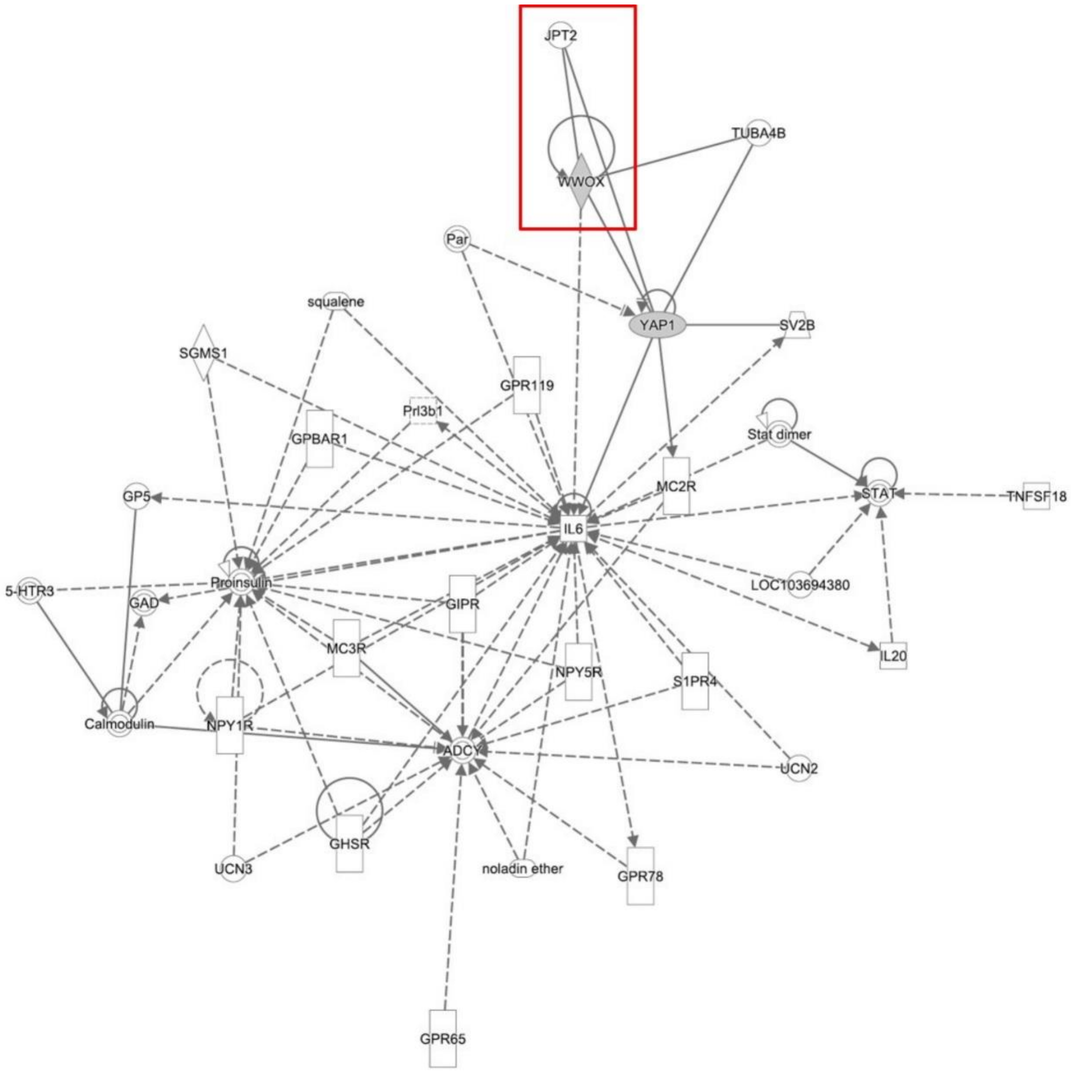

Figure S5. Ingenuity Pathway Analysis (IPA) was performed to find WWOX-interacting proteins. It is predicted that WWOX could interact with JPT2 (red box).

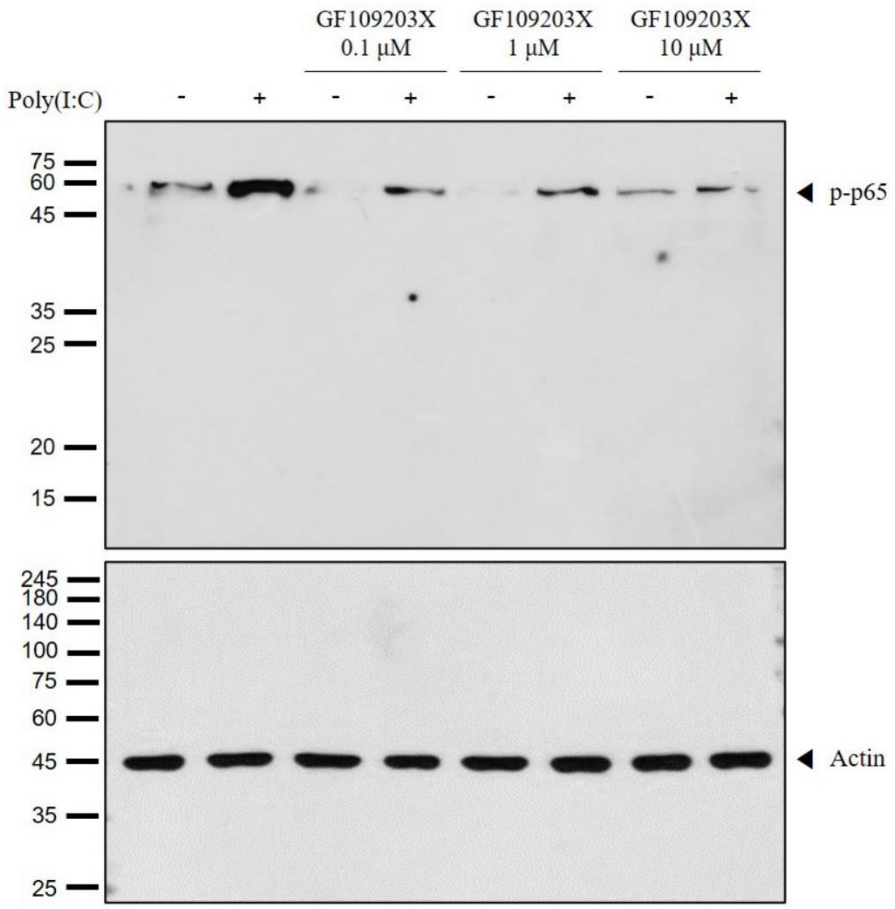

Figure S6. Uncropped data for Figure 5E.
